# Tumor specimen cold ischemia time impacts molecular cancer drug target discovery

**DOI:** 10.1038/s41419-024-07090-x

**Published:** 2024-09-26

**Authors:** Silvia von der Heyde, Nithya Raman, Nina Gabelia, Xavier Matias-Guiu, Takayuki Yoshino, Yuichiro Tsukada, Gerry Melino, John L. Marshall, Anton Wellstein, Hartmut Juhl, Jobst Landgrebe

**Affiliations:** 1 Indivumed GmbH, Hamburg, Germany; 2 Department of Pathology, Hospital Universitari Arnau de Vilanova, Universitat de Lleida, IRBLLEIDA, Lleida, Spain; 3 Department of Gastrointestinal Oncology, National Cancer Center Hospital East (NCCE), Kashiwa, Japan; 4 Department of Colorectal Surgery, National Cancer Center Hospital East (NCCE), Kashiwa, Japan; 5 Department of Experimental Medicine, University Tor Vergata, Rome, Italy; 6 The Ruesch Center for the Cure of Gastrointestinal Cancers, Georgetown University, Washington, DC USA; 7 Department Oncology & Pharmacology, Lombardi Comprehensive Cancer Center, Georgetown University, Washington, DC USA

**Keywords:** Cancer, Translational research

## Abstract

Tumor tissue collections are used to uncover pathways associated with disease outcomes that can also serve as targets for cancer treatment, ideally by comparing the molecular properties of cancer tissues to matching normal tissues. The quality of such collections determines the value of the data and information generated from their analyses including expression and modifications of nucleic acids and proteins. These biomolecules are dysregulated upon ischemia and decompose once the living cells start to decay into inanimate matter. Therefore, ischemia time before final tissue preservation is the most important determinant of the quality of a tissue collection. Here we show the impact of ischemia time on tumor and matching adjacent normal tissue samples for mRNAs in 1664, proteins in 1818, and phosphosites in 1800 cases (tumor and matching normal samples) of four solid tumor types (CRC, HCC, LUAD, and LUSC NSCLC subtypes). In CRC, ischemia times exceeding 15 min impacted 12.5% (mRNA), 25% (protein), and 50% (phosphosites) of differentially expressed molecules in tumor versus normal tissues. This hypoxia- and decay-induced dysregulation increased with longer ischemia times and was observed across tumor types. Interestingly, the proteomics analysis revealed that specimen ischemia time above 15 min is mostly associated with a dysregulation of proteins in the immune-response pathway and less so with metabolic processes. We conclude that ischemia time is a crucial quality parameter for tissue collections used for target discovery and validation in cancer research.

## Introduction

One of the major concepts in identifying functional proteins as oncogenic targets is the detection of alterations that lead to aberrant signaling in cancer-relevant pathways. A core principle of onco-pharmacological targeting approach is detecting and applying therapeutics that antagonize oncogenic protein function and thereby significantly improve overall survival [1]. An important strategy to systematically identify new target proteins for cancer treatment is to build a cancer registry combining tissue and metadata collection, a strategy pursued, for example, by the TCGA consortium (https://www.cancer.gov/tcga). To this end, cancer and ideally also matching normal adjacent tissue are collected and analyzed to compare the molecular characteristics of the two tissue types. The two parameters which have the strongest impact on the quality of tissue registries are the asservation method and the so-called cold ischemia time (in short ‘ischemia time’), which denotes the time it takes to freeze the tissue for permanent storage after its removal from the body during surgery.

There is an ongoing debate about the consequences of ischemia on the quality of biomolecules, especially in the context of characterizing diverse molecular entities of tissues or cells (so-called ‘omics’). The corresponding findings vary widely [2–4] in part due to insufficient numbers of samples and lack of annotation for ischemia time leading to an insufficient power to detect the impact of ischemia time on the molecular composition of the materials. Hence, the impact of ischemia time on the molecular characteristics of the tissue under investigation remains unclear. Here, we use a large set of samples annotated for ischemia time to investigate the impact on candidate target discovery.

We focus on the impact of ischemia time on differential expression of mRNAs, proteins and phosphoproteins using a carefully curated collection of patient-derived fresh-frozen tissue samples and related multiomic data. This database contains the breadth of molecular characteristics of tumor and normal adjacent tissues from multiple cancer types. Specimens were analyzed at the genomic, transcriptomic (TRX), proteomic (PTX) and phosphoproteomic (PPX) level with focus on colon cancer (CRC). Analyses were expanded to hepatocellular carcinoma (HCC) and non-small cell lung epithelial cancer (NSCLC), covering lung adenocarcinoma (LUAD) and lung squamous cell carcinoma (LUSC).

To generate a baseline in our studies we set an initial filter to capture biomolecules differentially expressed in the group of samples with the shortest ischemia time available (*<*10 min.). We then analyze the changes in expression of these biomolecules over time with an emphasis on the impact of ischemia time on the target identification process, rather than trying to model the tissue decay under ischemia.

We found that DNA sequences are to a large extent unaffected even by the longest ischemia times of samples collected. In contrast, the relationship between tumor and normal tissue mRNA, protein and phosphoprotein expression is affected at increasing severity (mRNA < protein < phosphoproteins). Based on the analyses we propose an ischemia time cut-off threshold at 12 min that enables a sufficient amount of tissues to be collected while avoiding a dilution of signals for biomolecules of interest.

## Results

### Cancer samples and differential expression

Datasets and samples used in the analysis are shown in Table 1, tumor stages are shown in Table 2. Details on the samples can be found in the Methods section.

**Table 1:** Number of tumor samples per ischemia time interval (min.) with specific omic data.

Cohort	*T*_1_: *t* < 10			*T*_2_: 10 ≤ *t* ≤ 12			*T*_3_: 13 ≤ *t* ≤ 15			*T*_4_: 16 ≤ *t* ≤ 18			*T*_5_: 19 ≤ *t* ≤ 20			*t* > 20			Total		
	TRX	PTX	PPX	TRX	PTX	PPX	TRX	PTX	PPX	TRX	PTX	PPX	TRX	PTX	PPX	TRX	PTX	PPX	TRX	PTX	PPX
CRC	181	188	188	223	223	217	111	102	101	44	47	47	18	19	19	36	54	54	613	633	626
HCC	48	41	41	35	36	36	11	9	9	15	15	16	6	6	6	32	38	38	147	145	146
LUAD	215	258	255	119	152	153	90	95	94	51	50	48	17	26	26	35	44	44	527	625	620
LUSC	134	157	153	95	103	102	56	58	58	40	37	35	20	23	23	32	37	37	377	415	408

**Table 2:** Number of tumor samples per cancer stage group with specific omic data.

	I			II			III			IV			NA			Total		
Cohort	TRX	PTX	PPX	TRX	PTX	PPX	TRX	PTX	PPX	TRX	PTX	PPX	TRX	PTX	PPX	TRX	PTX	PPX
CRC	65	60	60	229	251	248	195	201	199	121	118	116	3	3	3	613	633	626
HCC	31	33	34	18	22	22	18	17	17	7	7	7	73	66	66	147	145	146
LUAD	232	293	291	129	145	144	133	143	141	24	33	33	9	11	11	527	625	620
LUSC	126	151	147	125	130	130	116	119	116	6	8	8	4	7	7	377	415	408

We defined the ischemia time reference group as the group of samples with ischemia times less than 10 min, the shortest time possible for the collection and storage of a sufficient number of samples. To obtain differential biomolecule expression for mRNAs, proteins and phosphoproteins for the shortest ischemia time group, we normalized the expression data and selected the differentially expressed biomolecules using non-parametric Wilcoxon tests for paired samples to take into account the non-normal distribution of the data [5] as described in the Methods part. We adjusted the resulting *P*-values for multiple testing and selected the biomolecule sets for mRNA, protein, and phosphoprotein using an *α*_fdr_ level of 0.01 and an effect boundary using the 5th and 95th percentiles of the effect distribution. For the CRC cohort this yields 1948 differentially expressed mRNAs, 794 proteins, and 1846 phosphosites. Analogously, we identified 1870 differentially expressed mRNAs, 523 proteins, and 388 phosphosites in the HCC cohort. In the LUAD cohort, we found 1951 differentially expressed mRNAs, 805 proteins and 2217 phosphosites, and in the LUSC cohort, we found 1950 differentially expressed mRNAs, 798 proteins and 1919 phosphosites on which we focussed in the subsequent analyses.

### Differential expression over time in CRC

We performed a detailed analysis of differential (tumor versus normal adjacent tissue) expression over time in CRC, and then applied the most relevant analyses to the other cancer types listed in Table 1.

To evaluate whether DNA sequences remain unaffected by ischemia times as reported by others [6], we analyzed protein sequences for affecting mutations (PAM), the presence or absence of gene deletions or amplification as well as gene truncations by comparing the shortest available ischemia time tissue group with the longer ischemia time groups for each genomic locus (see Methods). At *α*_fdr_ = 0.01, only 0.09% of the proteins showed a signal in the PAM submodality, and no change was found in the other DNA-derived submodalities. This is likely based on random somatic difference in the genomic sequence of the patients of the various time groups (see section “Discussion”).

**Fig. 1:**
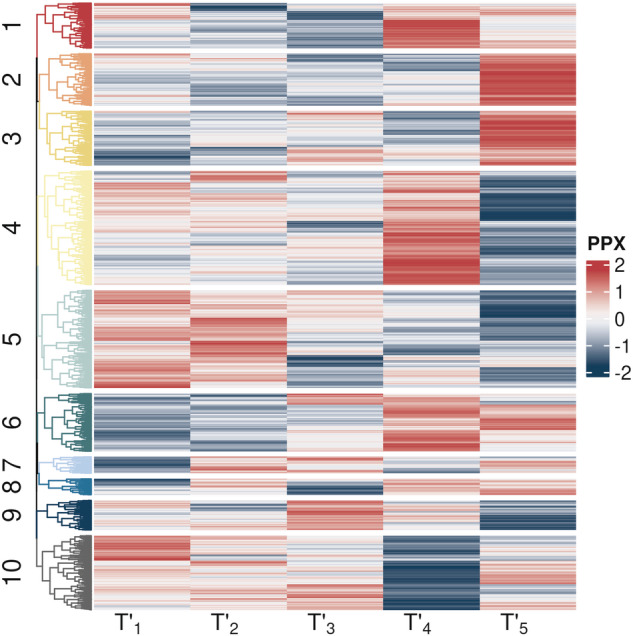
Differential expression of the selected phosphosites in 5-min intervals (abscissa) grouped into ten clusters (ordinate) in CRC. Colors indicate log_2_-fold mean expression differences (phosphosite-wise standardized) between tumor and normal tissue. Red: upregulation in tumor, blue: downregulation.

To obtain an overview of the effect of ischemia time on the selected biomolecules, we initially partitioned the samples into groups of *T*_1_: *t* < 10*, T*_2_: 10 ≤ *t* ≤ 14*, T*_3_: 15 ≤ *t* ≤ 19*, T*_4_: 20 ≤ *t* ≤ 24*, T*_5_: *t* ≥ 25 min of ischemia time. We then used hierarchical clustering to assess the changes in biomolecule expression for the three omic modalities as described in “Material and methods” section. Figure 1 shows the results for the phosphoprotein modality for 10 clusters (the corresponding plots for the other two expression modalities are shown in Supplementary Figure S1).

The effect magnitude and significance seem to increase for times over 20 min. To confirm this impression, we tested for differences in differential expression over time in each modality using a 2 × 5 factorial ANOVA-like design with the tissue types (tumor and normal) as first factor and the time groups *T*_1_ *… T*_5_ as second factor using the non-parametric Scheirer–Ray–Hare test also assessing interaction effects between the two main effects. Table 3A shows the phosphosites per cluster with significant tissue, time or interaction effects. The three effects on display are the two main effects of tissue and time difference and the interaction effect of the two variables.

**Table 3:** Alterations in phosphosite expression over time across tissue types.

A: Long duration ischemia time groups				B: Refined ischemia time groups			
Significant (*α*_fdr_ = 0.05) effect for				Significant (*α*_fdr_ = 0.05) effect for			
Cluster	Tissue	Time	Interaction	Cluster	Tissue	Time	Interaction
1a	147	45	0	1b	115	16	0
2a	169	63	3	2b	293	51	2
3a	177	61	6	3b	283	35	2
4a	369	129	2	4b	95	11	0
5a	317	101	12	5b	136	25	0
6a	188	68	8	6b	89	10	0
7a	54	11	2	7b	442	80	1
8a	52	16	0	8b	189	21	2
9a	97	35	0	9b	74	15	0
10a	242	76	6	10b	105	20	2
Totals	1812	605	39	Totals	1821	284	9

Table 3 confirms that across all clusters (note that the clusters in both panels do not contain identical phosphosites since the data vectors per phosphosite differ between the panels) of the original time groups (panel A), 33% (605/1814) of the phosphosites display a significant time effect. We therefore repartitioned the available samples into the shortest ischemia group (samples with ischemia time shorter than 10 min), and into further groups of 3 min intervals from *t* ≥ 10 to 20 min (*T*_1_ *… T*_5_) as shown in Table 1 in order to quantify which ischemia time point to use as cut-off (we call these *refined* time groups). With these refined groups (Table 3B) which exclude longer ischemia times, the time effect halves to 16% (284/1823) of the phosphosites. Note that the differing denominator between the two groupings is due to reassigned samples and a subsequently altered pattern of missing values. There are also fewer interaction effects when excluding longer ischemia times. The trends for mRNA and protein modalities are comparable, though less pronounced (see Supplementary Fig. S1 and Supplementary Tables S1 and S2). But even though the effect is least pronounced in mRNA, to perform valid multiomics analyses, we need an ischemia time limit that is the same for all modalities. Thus, we aim for a limit that is optimal for the most sensitive type of molecule (phosphate groups), which must be under 20 min.

Before investigating differential biomolecule expression, we analyzed the distribution of the underlying expression levels in normal and cancer tissue samples of the groups *T*_1_ *… T*_5_. We specifically focused on biomolecules with extreme expression values which could play an important role in cancer. We regarded biomolecules with expression values outside the (*Q*_2.5_*, Q*_97.5_) interval as extremely expressed. We determined the relative loss of such extreme biomolecules of reference group *T*_1_ in other ischemia time groups. In detail, we counted how many biomolecules with extreme expression values outside (*Q*_2.5_*, Q*_97.5_) of *T*_1_ were not detected outside the (*Q*_2.5_*, Q*_97.5_) intervals of the other groups *T*_2_ *… T*_5_ any more. For example for *T*_1_ and *T*_2_, this relative loss is

$${\mathcal L} =\frac{|\delta ({T}_{1})\backslash \delta ({T}_{2})|}{|\delta ({T}_{1})|}\cdot 100$$

where *δ* computes the biomolecules outside the interval (*Q*_2.5_*, Q*_97.5_) and | · · · | gives the set size. Table 4 shows the loss rates for tumor and normal tissues for the time group average. The vanishing biomolecules fall from outside (*Q*_2.5_*, Q*_97.5_) into the interval in the time groups *T*_2_ *… T*_5_.

**Table 4:** Loss L of biomolecules outside the (*Q*_2.5_*, Q*_97.5_) interval.

Modality	Tissue	Biomolecule loss [%] vs. *T*_1_			
		*T*_2_	*T*_3_	*T*_4_	*T*_5_
mRNA	Normal	27	32	39	21
	Tumor	23	28	45	27
Protein	Normal	24	25	36	56
	Tumor	20	23	27	42
Phosphoprotein	Normal	49	64	72	94
	Tumor	46	60	64	81

The loss of biomolecule sets in group *T*_2_ is striking for all types of analyzed biomolecules, but especially for phosphosites. Notably, the loss of the most highly expressed mRNAs is slower than for proteins and even more so than for phosphoproteins, where almost 50% of the most highly expressed sites are lost in a short interval time between 10 and 12 min of ischemia. In mRNA, there seems to be a recovery of the initial expression pattern after the longest ischemia duration, an artifact that may be explained by the degradation and altered detection of the molecules. This has to be taken into account when looking at differential biomolecule expression.

#### Temporal patterns of refined time series

To evaluate the influence of ischemia on differential expression patterns, we next analysed the shorter interval groups *T*_1_ *… T*_5_ using a Dirichlet process Gaussian process mixture model (DPGP, details see “Materials and methods” section). This non-parametric time series analysis technique jointly models data clusters with a Dirichlet process and temporal dependencies with a Gaussian process. Note that this is a pseudo-time series.

**Fig. 2:**
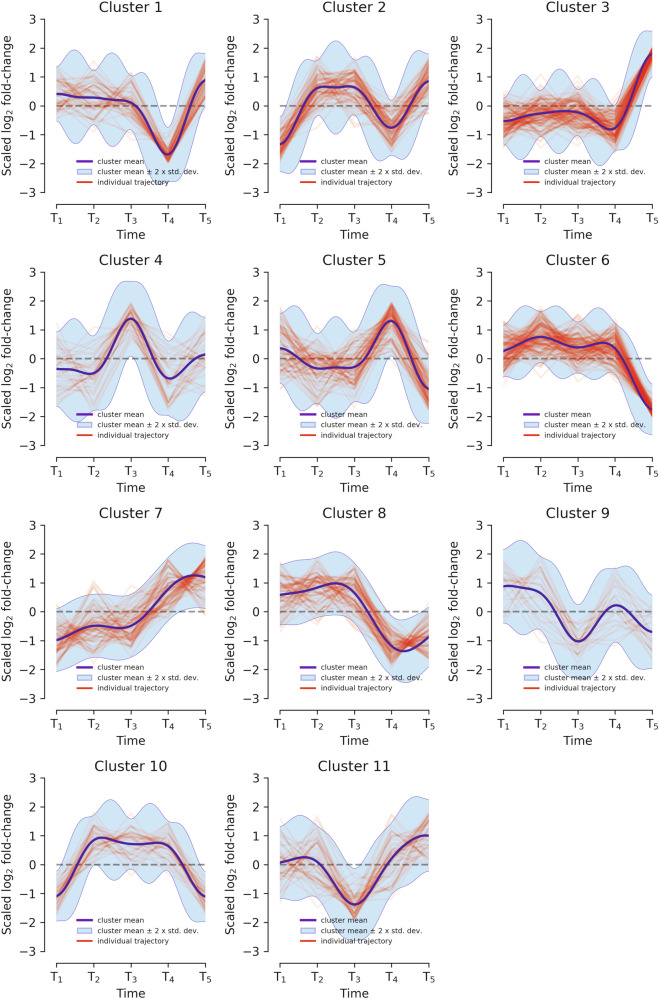
Differential expression of the selected proteins in refined time groups (*T*_1_*… T*_5_ as 3-min intervals on the abscissa) grouped into 11 clusters. The ordinates of each cluster show the normalized differential expression. The blue line shows the cluster mean expression. The red lines indicate the individual protein expression levels. The shaded blue area indicates the cluster mean ±2 · *σ*.

Figure 2 shows the eleven clusters obtained in CRC for the protein modality, which we selected out of the three available expression modalities because it allows for a deeper biological interpretation (analogous time series analyses were generated for each modality and tumor type but we did not gain additional biological insights).

We expect various patterns of protein expression variance due to ischemia with proteins losing or gaining abundance of expression due to the effect of the ischemia.

##### Biological cluster interpretation

To interpret the biological meaning of the protein expression patterns, we used a Wilcoxon test to identify proteins with differential ex- pression from one time group to another (*µ*_*T*2_ − *µ*_*T*1_,…, ${{\mu}}_{T_{k+1}}$ −${{\mu}_{{T}_{k}}}$, *k* = 2 *…* 4) excluding effects inside the interval (2^*−*1*/*2^, 2^1*/*2^).

We then mapped the genes related to these differentially expressed proteins to KEGG (https://www.genome.jp/kegg/mapper/search.html) and performed a pathway enrichment analysis. We could not find cluster-specific relevant biological patterns, maybe because ischemia regulation cannot be revealed by DPGP-clustering on our pseudo-time series, but we still found interesting overall patterns. The analysis revealed acute inflammatory response and metabolism as the most significantly up- and downregulated pathways, respectively. Table 5 shows the number of significantly differentially expressed proteins per pathway.

**Table 5:** Pathways with differential protein expression *T*_*k*+1_ − *T*_*k*_.

Pathways	Number of proteins	
	Upregulated	Downregulated
Immune response	9	2
Metabolic processes	5	8
Regulation of cell signaling	7	2
Transport of molecules	2	3
Cell structure and adhesion	1	2
Total	24	17

The data show an upregulation of proteins which are involved in immune-response but also in regulation of various cellular pathways and metabolism. Specifically, proteins involved in lipid metabolism are upregulated, namely ACSL4, FMO5, MOGAT2 and SULT2B1. That is most likely to supply for the high energy requirements induced by oxidative stress. Interestingly, 20% of the proteins in Table 5 are already deregulated between subsequent time points below 16 min (group *T*_2_ and *T*_3_), indicating that alterations can occur rapidly under ischemic conditions. All of the deregulated proteins and their expression profiles are listed in Supplementary Table S3. There is almost no differential expression effect in this group based on a tissue effect. These pathways reveal a coping mechanism involving a decrease in expression of proteins that are either not survival-critical under metabolic stress induced by ischemia (such as proteins involved in drug metabolism like UGT2B17 or UGT1A8) or those that are energy intensive for the cell (ribosome biogenesis). Ischemia very interestingly appears to slowly breach through this coping mechanism by causing a decrease in tumor survival-critical proteins such as NUDT1, ELOVL5, HPGDS or DZIP3 [7–10].

#### Confounder analysis

We next analyzed the influence of ischemia time on differential biomolecule expression compared to other independent variables of known clinical importance by performing a classical confounder analysis using multiple linear regression. We computed a multi-variate linear model with a Gaussian error distribution at the biomolecule level for each omic type and each of the differentially expressed biomolecule sets obtained from the initial selection step described in subsection “Cancer samples and differential expression”.

**Fig. 3:**
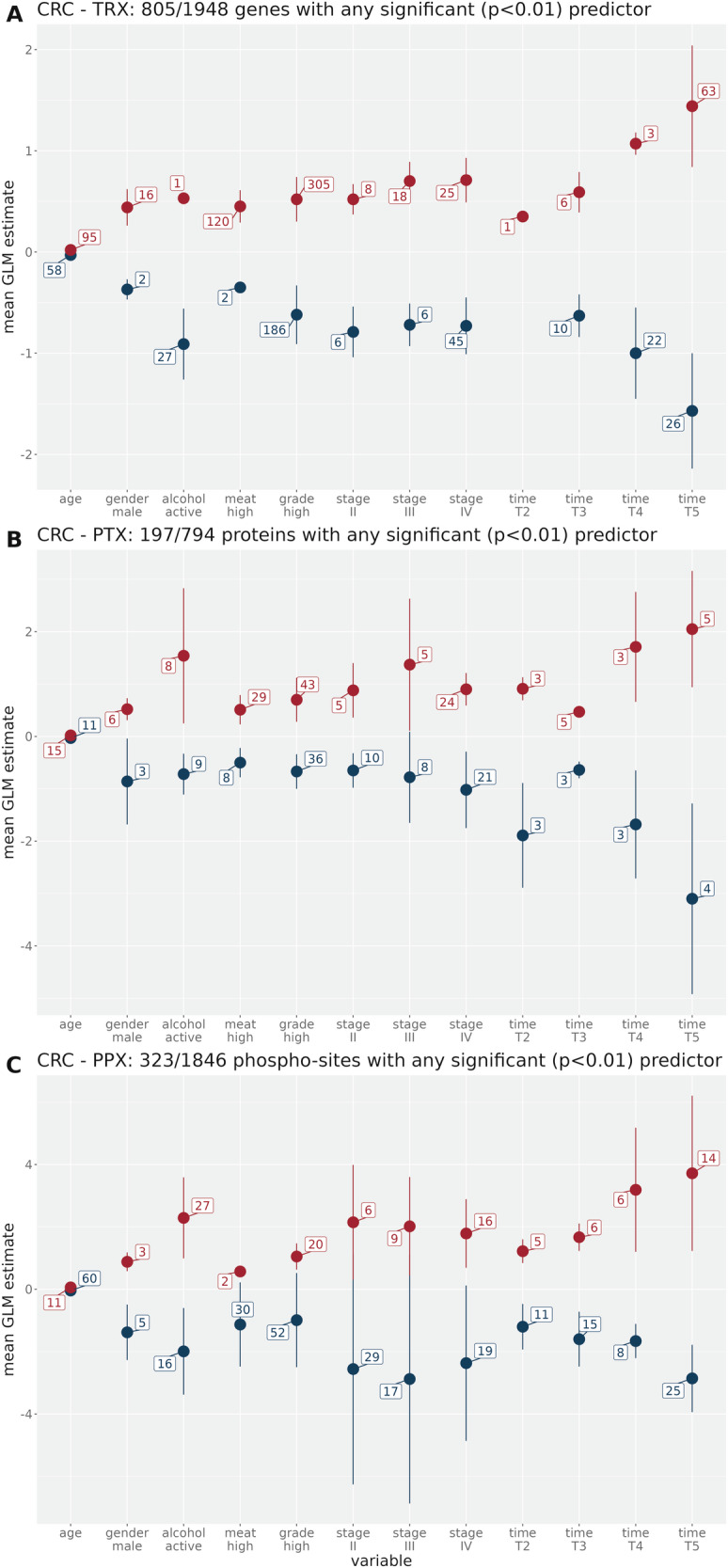
Mean regression coefficient estimates and standard deviation of important predictors (linear model T-statistic with *p* < 0.01) for the differentially expressed biomolecules in CRC. Numbers related to each variable denote the number of biomolecules with any significant positive (red) or negative (blue) effect. For categorical variables, the reference variables of the linear model are indicated in section 4. For example, *T*_1_ is the reference group for the other ischemia times. **A** Transcriptome, **B** Proteome, **C** Phosphoproteome.

The variation of the fold-change between tumor and normal tissue expression was modeled as dependent variable to be explainable by the independent variables (details see section “Material and methods“). This analysis is only a rough approximation because at the biomolecule level both statistical assumptions for Gaussian linear models of normal error distribution and linear variable relations are not fully met as shown by statistical testing (not shown). Nevertheless, the analysis revealed informative trends when we determined the distributions of variable effect estimates for biomolecules with any statistically significant (*p* < 0.01) variable. The corresponding plots of regression coefficient estimate means, split by positive and negative effects, for the CRC cohort are shown in Fig. 3. Note that variables which did not have any significant effect on any biomolecule are not shown in the confounder plots (details see section “Material and methods”).

The figure shows that for CRC, disregarding the ischemia time variable effects (for which *T*_1_ is the reference variable), the tumor grade and stage as well as the alcohol consumption status of the patients relative to their respective references are the strongest predictors of differential biomolecule expression as expected [11, 12]. High grade has not only high coefficient estimates, but also the highest number of biomolecules with significant effects in each modality (491, 79, and 72, for mRNA, protein and phosphosite, resp).

What about the effect of ischemia time? With the exception of protein expression, the ischemia group *T*_2_ shows the lowest *T*_1_-relative impact on the outcome compared to the other time groups, but overall, the influence of ischemia on differential biomolecule expression increases with longer ischemia times. If the ischemia time exceeds 12 min, the average coefficient estimate of this variable is at least as strong as the stage-IV estimates, and above 15 min it is much higher (also higher than grade). The number of biomolecules with significant *T*_5_ estimates also surpasses the number of biomolecules with significant stage-IV estimates, but does not exceed the number of biomolecules significantly affected by grade—which demonstrates the high relevance of this variable to explain the difference between tumor and normal tissue.

The ischemia time effects gain importance from transcriptome to proteome and are strongest in the phosphoproteome. But in all modalities, the magnitude of the ischemia time estimates wipes out the impact of grade and stage, which are clinically the most important predictors of cancer survival. Thus, this analysis provides important insights into the influence of ischemia time on biomolecule expression relative to standard clinical parameters.

As a next step, we analyzed the data to identify an ideal ischemia time cut-off.

### Ischemia time cut-off

**Fig. 4:**
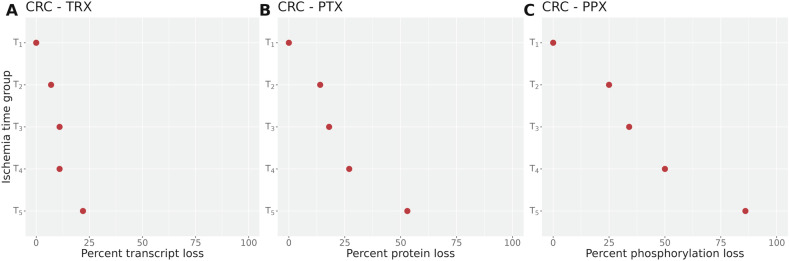
Relative loss of differentially expressed biomolecules in proportion to ischemia times in CRC. Dot plots showing the percentage of differentially expressed biomolecules exclusively detected in the shortest ischemia time group *T*_1_ (details see section 4) for each modality. The ordinate indicates the time group compared to *T*_1_, ordered from shortest to longest time interval, the abscissa indicates the percentage of biomolecule loss. **A** Transcriptome, **B** Proteome, **C** Phosphoproteome.

To find an ischemia time cut-off that optimizes the trade-off of collecting the maximum amounts of samples while not impeding the identification of differentially expressed biomolecules, we investigated how many biomolecules which are differentially expressed in the short ischemia time group (<10 min.) get lost with longer ischemia times. Figure 4 shows the relative biomolecule loss in the CRC cohort.

For the analysis, we computed the number of differentially expressed biomolecules for the three omic modalities, and found an increasing loss of deregulated biomolecules with longer ischemia times. At 20 min 22% of mRNAs, 53% of proteins, and 86% of phosphosites were lost in the analysis of the CRC cohort.

### Results for other cancer types

**Fig. 5:**
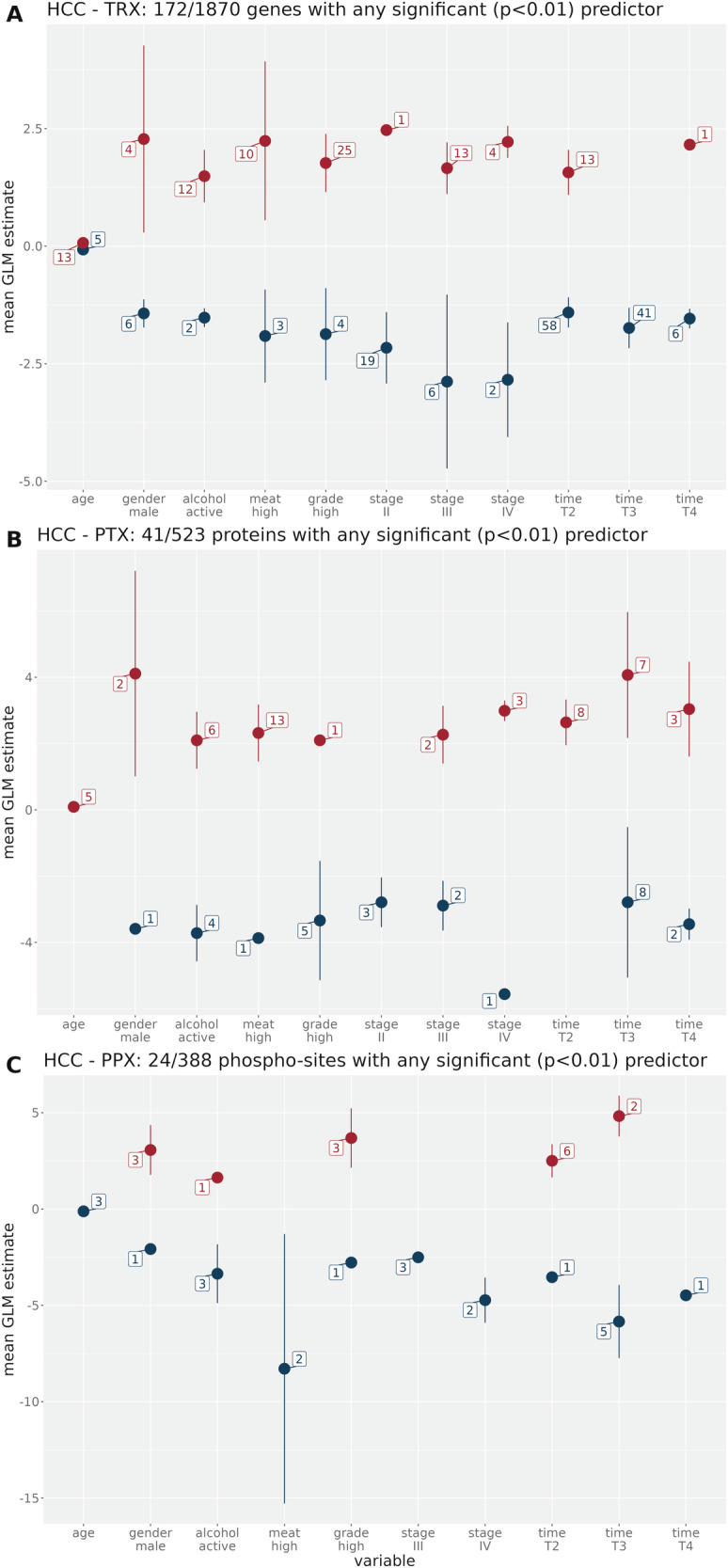
Mean regression coefficient estimates and standard deviation of important predictors (linear model T-statistic with *p* < 0.01) for the differentially expressed biomolecules in HCC. Numbers related to each variable denote the number of biomolecules with any significant positive (red) or negative (blue) effect. For categorical variables, the reference variables of the linear model are indicated in section 4. **A** Transcriptome, **B** Proteome, **C** Phosphoproteome.

We next evaluated the effects of ischemia time on differential biomolecule expression in HCC, LUAD, and LUSC tumor versus matching normal tissues. Because of small group sizes in *T*_3_ and *T*_5_ for HCC (see Table 1), we adjusted the time groups we used for the other cancer types in order to enable a reasonable statistical pseudo-time series analysis. Table 6 shows the adapted classification scheme which was applied in the HCC cohort; note that due to the different assignment to the groups, there is no group *T*_5_. Figure 5 shows the confounder analysis for HCC.

**Table 6:** Number of tumor samples per adapted ischemia time interval (min.) with specific omic data for HCC.

TRX	PTX	PPX
*T*_1_: *t* < 10		
48	41	41
*T*_2_: 10 ≤ *t* ≤ 12		
35	36	36
*T*_3_: 13 ≤ *t* ≤ 18		
26	24	25
*T*_4_: 19 ≤ *t* ≤ 30		
23	28	28
*t* > 30		
15	16	16
Total		
147	145	146

Interestingly, unlike in CRC, the ischemia estimates do not surpass those of the important clinical predictors in any modality in general, though more biomolecules tend to be significantly affected by ischemia time than by stage or grade. As in CRC, the effects are more pronounced for protein and phosphoprotein modalities than for the transcriptome. In the biomolecule loss analysis for HCC (Supplementary Fig. S4), the *T*_4_ group does not show a stronger loss of deregulated proteins or phosphosites compared to the earlier *T*_3_ group. This may be due to smaller sample number for HCC. Nevertheless, there is an analogous trend of losing differentially expressed biomolecules from *T*_1_ to *T*_3_.

The confounder analysis in LUAD (Supplementary Fig. S2) shows a pattern similar as CRC. Over time, the ischemia effect gains the same importance as the effect of the worst stage (mRNA) or surpasses it (protein and phophoprotein). The biomolecule loss in LUAD is comparable to CRC as well (see Supplementary Fig. S5).

The confounder analysis in LUSC (Supplementary Fig. S3) also shows the ischemia effect gaining importance over the clinical predictors with longer time, but in the protein and phosphoprotein modalities, there is an interesting exception, the stage-IV variable. In late-stage groups of LUSC differential biomolecule expression is more pronounced than in any other predictor group. We did not observe this in the other cancer types, though tumor grade is a very strong predictor in HCC for differential phosphosite expression. The biomolecule loss in LUSC is comparable to CRC and LUAD as well (see Supplementary Fig. S6).

## Discussion

There is an ongoing debate about the influence of ischemia time on data from various high-dimensional molecular characterization modalities (‘omics’) used in the analysis of cancer tissues. This discussion is important because these data are the foundation of the identification of indicators of disease outcomes (prognosis) and uncovering of targets for cancer treatment.

According to some authors, ischemia times of 30–60 min can be tolerated still yielding acceptable gene expression data [2] (*n* = 6 samples). Others reported that most phosphoproteins are stable over time [3] (*n* = 3 samples) or that RNA does not degrade for two hours at room temperature [4] (*n* = 18 samples). All of these studies included only small numbers of samples which will lead to low statistical power given the high dimensionality of molecular entities. On the other hand, several studies have shown that longer ischemia times reduce the quality of the measured molecular characteristics such as mRNA, protein, and phosphoprotein significantly [13–18]. However none of these studies have been able to use sufficient numbers of samples to define a cut-off value for detecting the impact of ischemia time on the molecular composition of the materials, in particular for multiomics data-driven target discovery.

Our research demonstrates the impact of ischemia time on the relative and absolute quantities and properties of DNA, mRNA, protein and phosphoprotein molecules using a large tissue sample collection between 145 (HCC) and 633 cases (CRC) of four different cancer types in total including matching adjacent normal tissue. Because our aim is to understand the impact of ischemia time on differential biomolecule expression, we first established a set of differentially expressed biomolecules at the shortest ischemia time group *T*_1_, which most closely mimics the in vivo status. We used this baseline to map changes in these biomolecules over time.

We first consider the underlying changes of the absolute biomolecule expression in normal and tumor tissue under ischemic conditions. Table 4 shows the drastic loss in expression levels of biomolecules among the 5% least or most strongly expressed mRNAs, proteins and phosphosites separately for tumor and normal tissue. One can imagine the decomposition of molecules in cells dying from ischemia as a highly chaotic non-ergodic complex process. Fundamentally, the decay process is comparable in both tumor and normal tissues as is also evidenced by the low proportion of interaction effects (Table 3B). Therefore, the differential biomolecule expression, which compares tumor and normal tissue expression, is less affected by ischemia than the individual expression levels per tissue type.

The data in Fig. 4 show that the effect of ischemia time on differential mRNA expression is weaker than for proteins and phosphoproteins. This pattern is observed across the different cancer types suggesting a more homogeneous mRNA decomposition between the tissue types.

Our main findings on the influence of ischemia time on differential biomolecule expression in the CRC cohort differ between the specific omic modalities. First of all, we do not see any effect on genomic DNA, as is expected from the biochemical properties of this molecule type which can even be recovered from paleontological fossils to obtain genetic sequences.

For the other modalities, as we see for phosphoproteins (Fig. 1 and Table 3), but also for mRNAs (Supplementary Table S1) and proteins (Supplementary Table S2), ischemia times over 25 min make analyses of rapidly decaying molecules unreliable.

Regarding the impact of the tissue type on biomolecule expression, i.e. tumor vs. normal tissue, and the ischemia time group or the combination of both variables (interaction effect), the tissue effect dominates the results since the biomolecules were selected based on group *T*_1_. Together with group *T*_2_ (10 ≤ *t* ≤ 12), which overall has an expression pattern very similar to *T*_1_ at least for mRNA and proteins, these time groups cover roughly ^2^*/*3 of the samples (cf. Table 1), hence dominating the main effect on biomolecule expression. The time and interaction effects in both panels of Table 3 reflect the influence of ischemia time on differential biomolecule expression. In our tissue collection, only ^1^*/*3 of the samples have longer ischemia times and their impact on expression observed at the shorter times is limited. Thus we do not see too many biomolecules with time effects due to the high quality of our tissue collection.

### Limitations of this study

#### Patient samples versus animal models of human cancer

The overall aim of our biobank is the identification of new targets for the treatment of human cancer. Thus, we focus on the analysis of human samples that is challenging due to the heterogeneity and complexity of diseased tissue. The central question addressed here is: What is the impact of ischemia time on the detectability of biomolecules in human cancer and matching normal tissue samples? Animal models of human cancer can provide valuable insights in cancer research. However, an analysis of samples from animal models would have been a poor substitute for the current analysis, with limited capacity to extrapolate to human samples.

#### Pathophysiology of ischemia

Cancer tissues with genomic instability, defective DNA repair, dysregulated gene expression and altered pathway activation react to hypoxia in quite distinct way compared to normal cells [19]. Here we do not attempt to address the molecular pathophysiology of hyopxia and ischemia in normal and cancer tissue, but the study merely aims to analyze the impact of ischemia on the ability to uncover potential targets for cancer treatment.

## Conclusions

Our omics analysis of tissues collected with different ischemia times reveals the complex changes of omics patterns. This finding challenges the interpretation and conclusions drawn from differential expression of cancer and normal tissues without proper accounting for ischemia time. The most clear pattern we observe across the time series clusters relates to immune-response proteins and metabolic function bearing proteins.

To reveal independent variables which could dominate differential expression in addition to ischemia time, we performed a systematic confounder analysis. As Fig. 3 shows, increasing ischemia times even surpass the effect estimators of alcohol consumption, grade, and cancer stage in the CRC cohort, which are known to strongly affect biomolecule expression in cancer tissue [11, 12]. However, the number of biomolecules for which the grade-covariable is significantly correlated to differential expression is not surpassed by the ischemia effect. While this can be related to the composition of our cohort with relatively few samples with longer ischemia times, the confounder analysis highlights the biological importance of grade, a predictor that is debated and sometimes underestimated in clinical practice [20].

The results obtained from lung cancers (LUAD and LUSC) are very similar to CRC. The number of biomolecules whose differential expression is significantly correlated to grade is also high in these cancer types.

Interestingly, in HCC the influence of ischemia time on the differential expression outcome is weaker than in the adenocarcinoma. However, as shown in the loss analysis (see Fig. S4), there is a considerable loss of differentially expressed biomolecules in hepatic tissue was well.

In summary, our experiments show that ischemia times below 12 min are recommended to obtain optimal differential biomolecule expression data. If samples with longer times are still to be included for specific reasons, they should be limited to a small proportion of the collection in order to obtain data of relevant scientific value.

## Material and methods

### Tissue sources and preparation

Indivumed GmbH has a tissue collection of fresh-frozen tumor samples with matching normal tissues. These samples were frozen after different ischemia times after removal from the situs of surgery.

Tissue samples were collected by Indivumed’s clinical partners using a standardized, IRB-approved protocol, focusing on minimal ischemia time. Our resection protocols enforce a completely analogous treatment of tumor and normal sample, the ischemia times do not differ. They were processed and pathologically assessed as previously described [21]. Nucleic acid extraction, library preparation and NGS were performed as previously described [21]. Protein extraction and MS analysis were performed as previously described [22, 23].

The data for CRC with matching colon mucosa from the same patient include 613 TRX (mRNA), 633 PTX (protein) and 626 PPX (phosphoprotein) datasets, for HCC with matching normal liver tissues 147 mRNA, 145 protein and 146 phosphoprotein datasets, and for LUAD 527 mRNA, 625 protein and 620 phosphoprotein datasets and for LUSC 377 mRNA, 415 protein and 408 phosphoprotein datasets with matching normal lung epithelium analyses.

Tumor stages, which are relevant for the confounder analysis we performed (see Fig. 3 and corresp. text), match the expected distribution for colon cancer surgery specimens (10% stage I, 33% II, 33% III and 20% IV). The proportions in HCC are roughly 4 : 2.5: 2.5 : 1 for stages I to IV, and the proportions for LUAD are similar. For LUSC, they are roughly 3:3:3:1, in between CRC and HCC proportions.

### Data processing and analysis

#### Processing and normalization

For DNA sequences obtained from whole genome sequencing, we analyzed protein amino-acid sequence affecting somatic mutations (PAM, counts per locus), copy number variations (binomial distribution per genomic locus for amplification or deletion, in two matrices resp.), and the presence of truncations (binomial data). A Kruskal*–*Wallis test was used to identify genes with a significant mutation load on the PAM count data, Fisher’s exact test was used for this purpose on the binomial data. For each genomic locus, the resulting *P* value for each ischemia time interval was compared to the data of the shortest time interval. For mRNA sequencing data, we adjusted batch effects of sequencing providers using ComBat-seq [24]. The normalized counts were then transformed to standard TPM values (transcripts per million) accounting for the length of the transcripts as described in [25]. Protein MS/MS signals were transformed to counts using DIA-NN [26], phosphoprotein MS/MS using Spectronaut 13 (Biognosys). For both modalities, a median normalization was performed per analysis run to scale the intensity values and make the runs comparable [27].

This preprocessing and normalization results for each cancer type (CRC, HCC, and NSCLC) in three matrices, i.e. one matrix per omic data type (mRNA, protein and phosphoprotein), with biomolecules (genes, proteins, phosphosites) in rows and samples in columns. When needed, the expression values of the paired samples were aggregated to log_2_-fold changes (tumor versus normal tissue), yielding matrices with half the number of columns.

#### Statistical analysis

Original code for the analyses can be obtained from the corresponding author upon request. For all statistical tests, the data met their assumptions if not otherwise indicated and justified. The reported statistics take into account variance estimates within and between groups.

*Differential biomolecule expression* was calculated for the short ischemia time group *T*_1_ using the two-sided Wilcoxon test for paired samples. P-values were adjusted for multiple testing via the method of Benjamini and Hochberg [28] which was the default correction method if not reported otherwise. The effect was computed as the mean difference of the log_2_ transformed expression values between tumor and normal tissue. To assess differences in differential expression between tissue types (tumor versus normal) across ischemia time groups, we used the *non-parametric Scheirer–Ray–Hare* test for 2-factorial designs with the normalized expression values as outcome.

To perform *hierarchical clustering* on the differentially expressed biomolecules in the short ischemia time group *T*_1_, for each modality the matrix of mean log_2_-fold changes (tumor versus normal tissue) was row-wise normalized (mean-centered and scaled by dividing by standard deviation). We then applied hierarchical clustering with complete linkage (using the Euclidean distance metric:

$$d({x}_{i},{y}_{i})={\Vert {y}_{i}-{x}_{i}\Vert }_{2}=\sqrt{{\sum }_{j=1}^{p}{({y}_{ij}-{x}_{ij})}^{2}}$$

with *x*_*i*_ and *y*_*i*_ two matrix rows and *j* = 1*…P* the matrix columns) to the matrix rows and displayed the results as heatmaps with dendrograms. The number of clusters was determined by visual inspection. The clusters were used to refine the time groups and limit them to 20 minutes; they are merely illustrative and irrelevant for the further analyses.

For the *clustering of these deregulated biomolecules in the refined ischemia time groups*, we used a Dirichlet process (DP) Gaussian process (GP) mixture model [29]. This non-parametric time series analysis technique jointly models data clusters with a Dirichlet process and temporal dependencies with Gaussian processes. The DPGP software (https://github.com/PrincetonUniversity/DP GP cluster) was parameterized with concentration parameter *α* = 0.1, the number of empty clusters in each iteartion *m* = 12, and the shape and scale parameters of the inverse gamma distribution set to *α*^*IG*^ = 4 and *β*^*IG*^ = 2, respectively.

The *confounder analysis* was performed by computing biomolecule-wise linear models with Gaussian error distribution for each modality using the differential biomolecule expression as response variable and *age*, *gender*, *alcohol consumption* and *red* *meat consumption* anamnesis, *histological grade*, *tumor stage* and the *refined ischemia time* groups as independent predictor variables. The values of the resulting effects as average changes in the log odds of the response variable associated with a one unit increase in each predictor variable were visualized, also including standard deviation, for the biomolecules with at least one significant predictor variable (t-statistic derived *p* < 0.01). Apart from the numeric continuous variable *age*, all other predictor variables were categorical ones with the following factor levels:

- *gender* : female (reference), male,
- *alcohol consumption*: inactive (reference), active,
- *meat consumption* (days per week): low (0-3 days, reference), high (4-7 days),
- *histological grade*:low (G1-G2, reference), high (G3-G4),
- *tumor stage*: I (reference), II, III, IV,
- *refined ischemia time*: *T*_1_ (reference), *T*_2_, *T*_3_, *T*_4_, *T*_5_.

The optimal *ischemia time cut-off* was inferred within a set-difference analysis for the refined time groups. This *set-difference analysis* was based on the results of the differential expression analysis. In detail, we identified the significantly differentially expressed biomolecules per time group *T*_*i*_*, i* = 1 *…* 5 (*α*_fdr_ = 0.01). We then determined the set differences {*T*_1_\*T*_*i*_ } of deregulated biomolecules in the shortest ischemia time group and the other groups. The percentage of the number of biomolecules in the set differences in relation to the number of differentially expressed biomolecules in the shortest ischemia time group was visualized in dot plots.

## Supplementary information

All supplementary figures and tables in one file

## Data Availability

The datasets generated during and/or analyzed during the current study are available from the corresponding author on reasonable request.
