# Supplementary material for: Tumor specimen cold ischemia time impacts molecular cancer drug target discovery
Source: Cell Death Dis. 2024 Sep 26;15(9):691. doi: 10.1038/s41419-024-07090-x (PMC11427669; doi:10.1038/s41419-024-07090-x)
Supplement: Supplementary file 1 — All supplementary figures and tables in one file [file 41419_2024_7090_MOESM1_ESM.pdf]

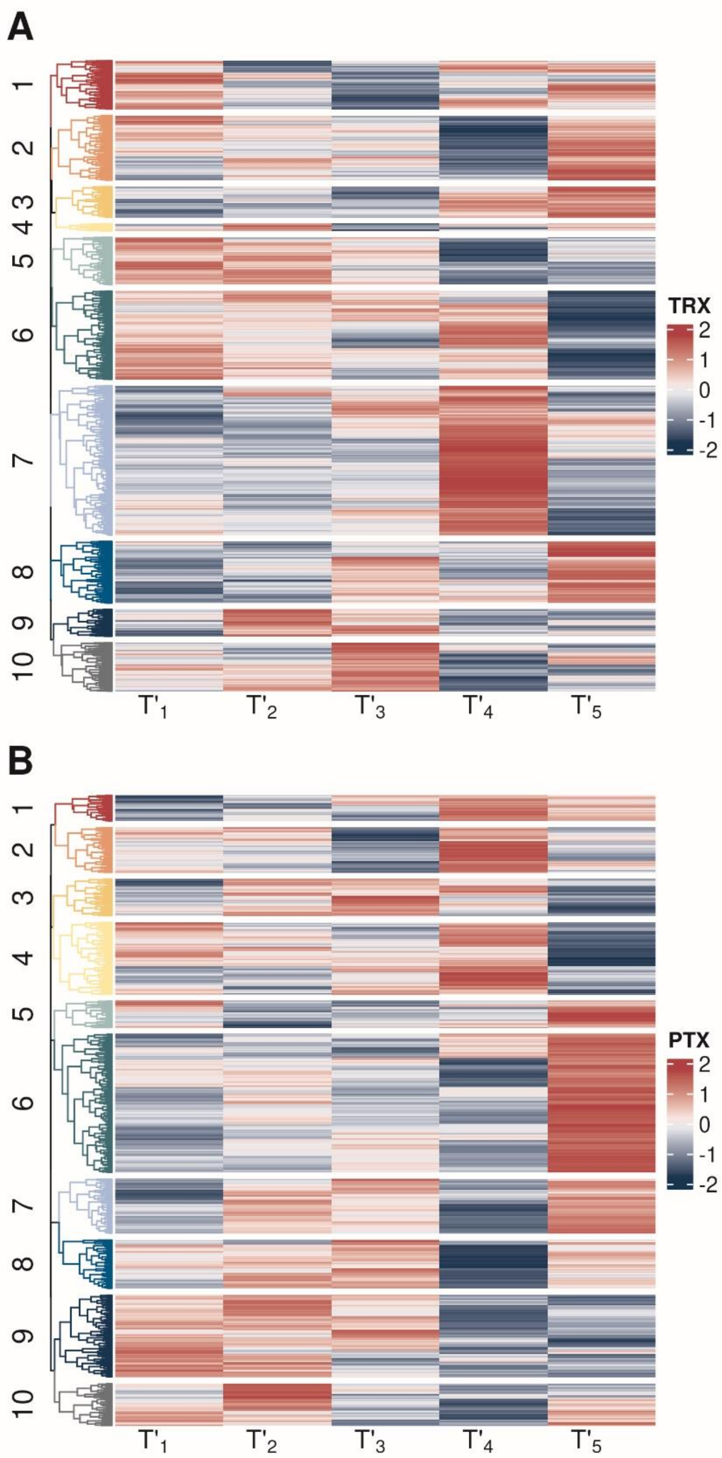

Supplementary Figure S1: Differential expression of the selected biomolecules in 5-minute intervals (abscissa) grouped into ten clusters (ordinate) in CRC. Colours indicate $\log_2$-fold mean expression differences (biomolecule-wise standardised) between tumour and normal tissue. Red: upregulation in tumour, blue: downregulation. **(A)** Transcriptome, **(B)** Proteome.

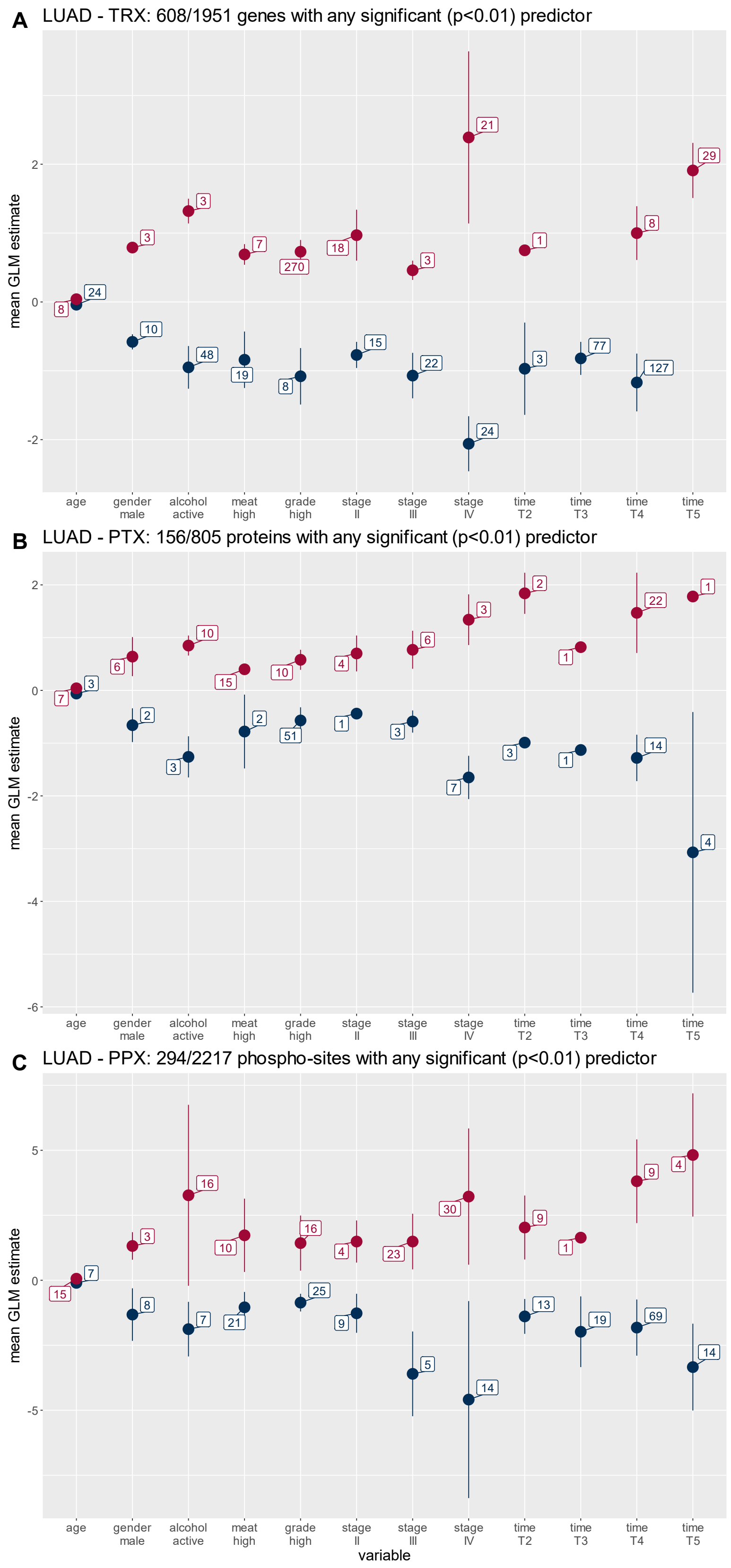

Supplementary Figure S2: Mean regression coefficient estimates and standard deviation of important predictors (linear model T-statistic with p < 0.01) for the differentially expressed biomolecules in LUAD. Numbers related to each variable denote the number of biomolecules with any significant positive (red) or negative (blue) effect. For categorical variables, the reference variables of the linear model are indicated in section 4. **(A)** Transcriptome, **(B)** Proteome, **(C)** Phosphoproteome.

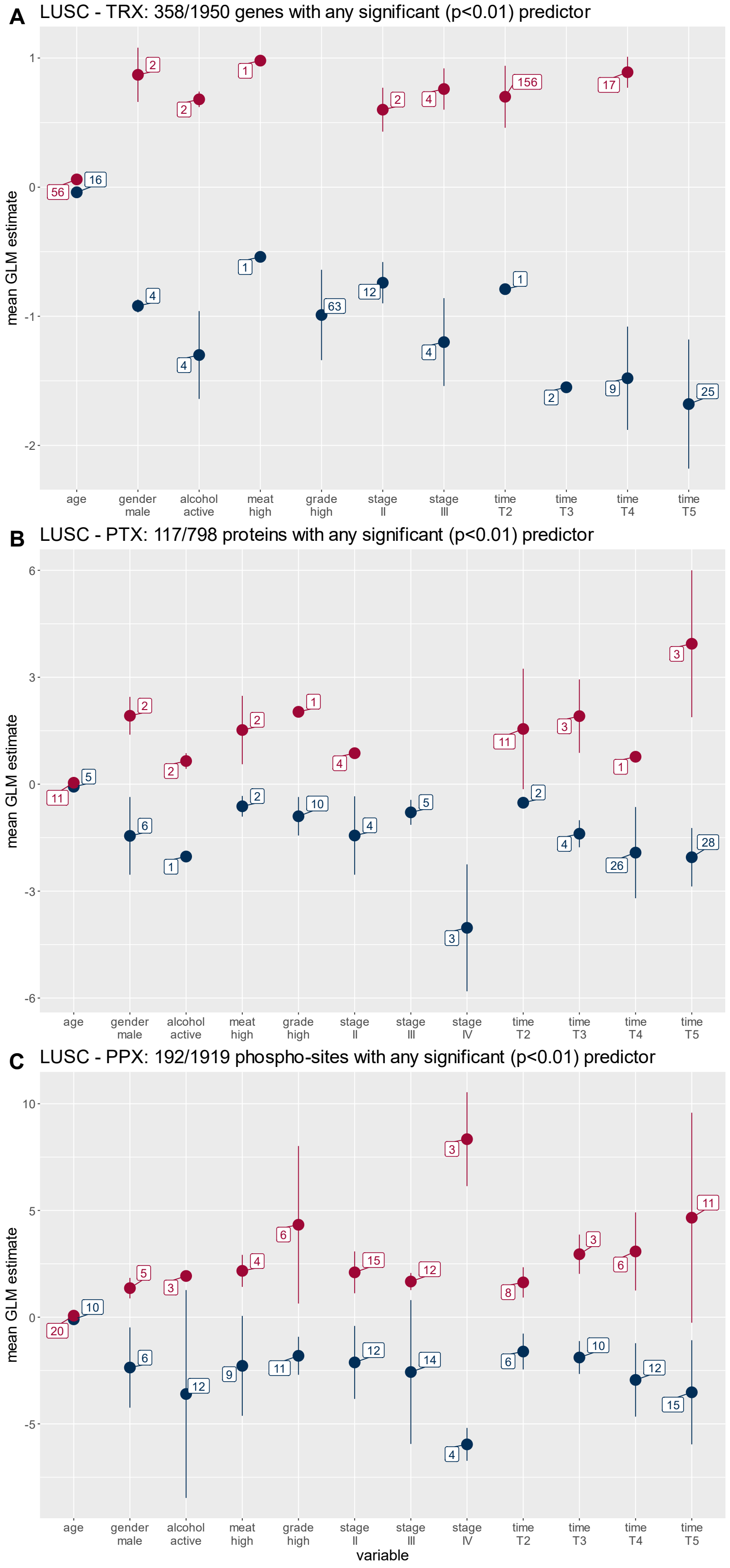

Supplementary Figure S3: Mean regression coefficient estimates and standard deviation of important predictors (linear model T-statistic with p < 0.01) for the differentially expressed biomolecules in LUSC. Numbers related to each variable denote the number of biomolecules with any significant positive (red) or negative (blue) effect. For categorical variables, the reference variables of the linear model are indicated in section 4. **(A)** Transcriptome, **(B)** Proteome, **(C)** Phosphoproteome.

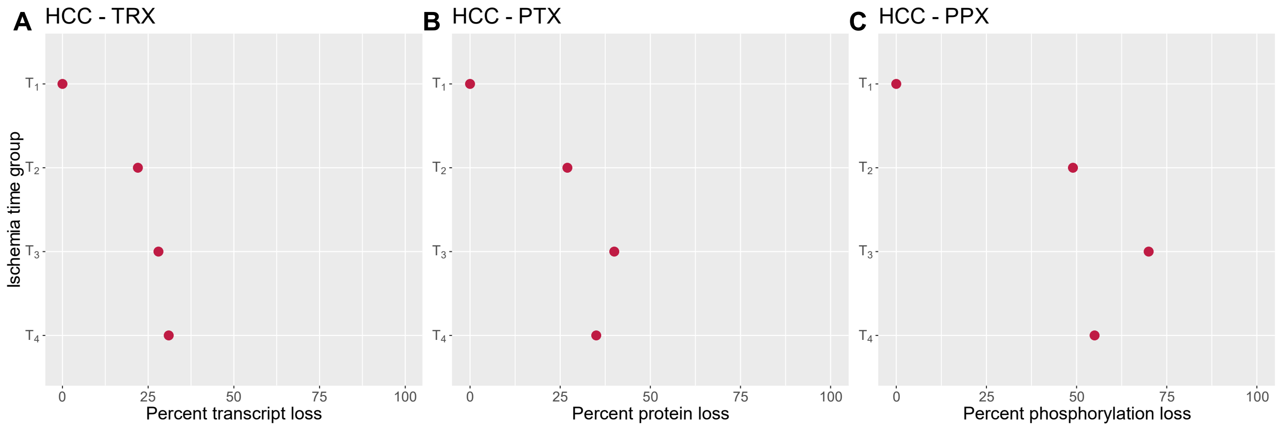

Supplementary Figure S4: Relative loss of differentially expressed biomolecules in proportion to ischemia times in HCC. Dot plots showing the percentage of differentially expressed biomolecules exclusively detected in the shortest ischemia time group T_1 (details see section 4) for each modality. The ordinate indicates the time group compared to T_1, ordered from shortest to longest time interval, the abscissa indicates the percentage of biomolecule loss. **(A)** Transcriptome, **(B)** Proteome, **(C)** Phosphoproteome.

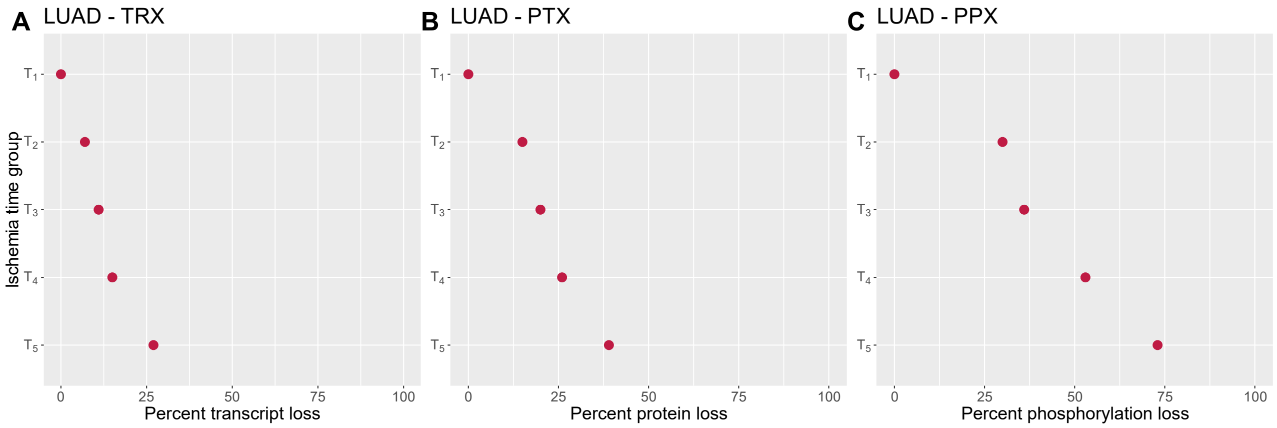

Supplementary Figure S5: Relative loss of differentially expressed biomolecules in proportion to ischemia times in LUAD. Dot plots showing the percentage of differentially expressed biomolecules exclusively detected in the shortest ischemia time group T_1 (details see section 4) for each modality. The ordinate indicates the time group compared to T_1, ordered from shortest to longest time interval, the abscissa indicates the percentage of biomolecule loss. **(A)** Transcriptome, **(B)** Proteome, **(C)** Phosphoproteome.

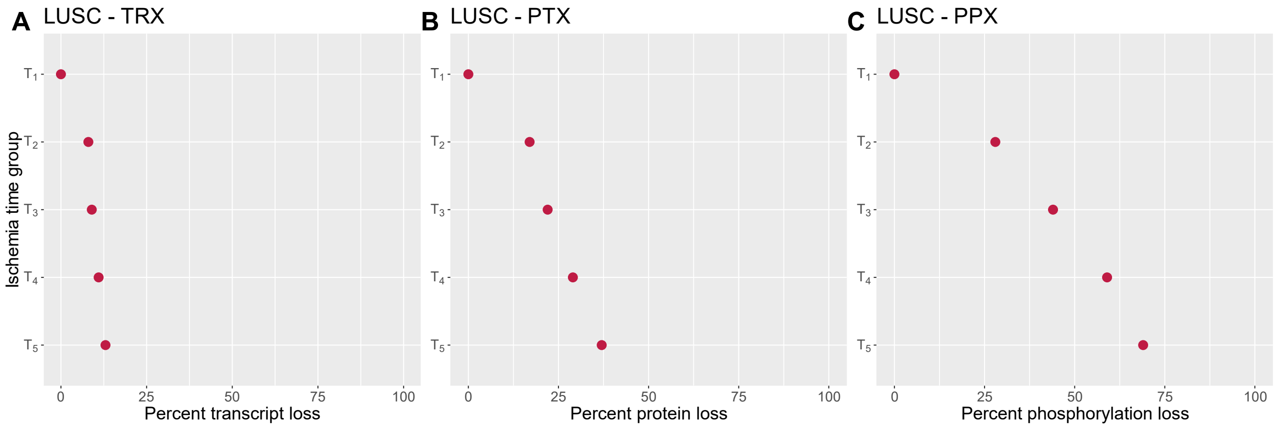

Supplementary Figure S6: Relative loss of differentially expressed biomolecules in proportion to ischemia times in LUSC. Dot plots showing the percentage of differentially expressed biomolecules exclusively detected in the shortest ischemia time group T_1 (details see section 4) for each modality. The ordinate indicates the time group compared to T_1, ordered from shortest to longest time interval, the abscissa indicates the percentage of biomolecule loss. **(A)** Transcriptome, **(B)** Proteome, **(C)** Phosphoproteome.

Table S1: Alterations in gene expression over time across tissue types

**A: Long duration ischemia time groups**				**B: Refined ischemia time groups**			
	Significant ($\alpha_{\text{fdr}} = 0.05$) effect for				Significant ($\alpha_{\text{fdr}} = 0.05$) effect for		
Cluster	Tissue	Time	Interaction	Cluster	Tissue	Time	Interaction
1a	165	7	0	1b	178	4	0
2a	220	10	0	2b	67	0	0
3a	105	2	0	3b	33	0	0
4a	25	0	0	4b	305	6	0
5a	161	3	0	5b	177	3	2
6a	300	4	0	6b	44	1	0
7a	506	7	1	7b	81	1	0
8a	209	6	1	8b	136	2	0
9a	92	0	1	9b	745	6	2
10a	165	4	0	10b	182	3	0
Totals	1948	43	3	Totals	1948	26	4

Table S2: Alterations in protein expression over time across tissue types

**A: Long duration ischemia time groups**				**B: Refined ischemia time groups**			
	Significant ($\alpha_{fdr} = 0.05$) effect for				Significant ($\alpha_{fdr} = 0.05$) effect for		
Cluster	Tissue	Time	Interaction	Cluster	Tissue	Time	Interaction
1a	36	5	0	1b	49	0	0
2a	62	5	0	2b	138	0	0
3a	51	2	0	3b	44	1	0
4a	99	3	0	4b	126	2	0
5a	37	3	0	5b	76	2	1
6a	190	16	6	6b	88	1	0
7a	75	8	0	7b	70	0	1
8a	67	9	0	8b	110	2	0
9a	113	10	0	9b	33	1	0
10a	58	3	0	10b	53	1	0
Totals	788	64	6	Totals	787	10	2

Table S3: Annotation of the significantly deregulated proteins between subsequent time groups (T_{k+1} vs. T_k, $k = 1 \ldots 4$) in DPGP clusters as summarised in Table 5

Cluster	Time T_{k+1}	Protein (HGNC)	Pathway	Deregulation (T_{k+1} vs. T_k)	Mean $\log_2$ fold-change (Tumour vs. Normal)
6	T_4	CNTN1	cell structure and adhesion	down-regulation	-2.19
6	T_5	KRT75	cell structure and adhesion	down-regulation	-6.57
9	T_2	DES	cell structure and adhesion	up-regulation	-1.98
1	T_4	IGLV4-69	immune response	down-regulation	-1.08
8	T_4	ADAMDEC1	immune response	down-regulation	-1.54
1	T_5	IGLV4-69	immune response	up-regulation	-1.08
3	T_5	CHGA	immune response	up-regulation	-2.84
3	T_5	IGHV3-72	immune response	up-regulation	-0.62
3	T_5	IGKV3-15	immune response	up-regulation	-0.53
3	T_5	IGKV3-20	immune response	up-regulation	-0.60
3	T_5	IGKV3D-15	immune response	up-regulation	-0.76
3	T_5	IGLV1-40	immune response	up-regulation	-0.96
3	T_5	RNASE6	immune response	up-regulation	-1.04
7	T_4	ISG15	immune response	up-regulation	1.49
1	T_3	UGT1A8	metabolic processes	down-regulation	-1.96
1	T_3	UGT2B17	metabolic processes	down-regulation	-3.23
6	T_5	DDX27	metabolic processes	down-regulation	0.79
6	T_5	FTSJ3	metabolic processes	down-regulation	0.72
6	T_5	HPGDS	metabolic processes	down-regulation	-2.09
6	T_5	NUDT1	metabolic processes	down-regulation	0.66
11	T_3	DZIP3	metabolic processes	down-regulation	0.79
11	T_3	ELOVL5	metabolic processes	down-regulation	0.45
3	T_5	ADH1B	metabolic processes	up-regulation	-2.41
3	T_5	FMO5	metabolic processes	up-regulation	-0.92
3	T_5	MOGAT2	metabolic processes	up-regulation	-0.85
7	T_4	ACSL4	metabolic processes	up-regulation	1.22
7	T_4	SULT2B1	metabolic processes	up-regulation	2.23
8	T_4	TPPP3	regulation of cell signalling	down-regulation	-1.88
11	T_3	SST	regulation of cell signalling	down-regulation	-4.11
3	T_5	GUCA2A	regulation of cell signalling	up-regulation	-1.07
3	T_5	TRIP13	regulation of cell signalling	up-regulation	1.87
3	T_5	VIP	regulation of cell signalling	up-regulation	-1.70
7	T_4	NOC2L	regulation of cell signalling	up-regulation	1.60
7	T_4	PYY	regulation of cell signalling	up-regulation	-2.92
7	T_5	DACH2	regulation of cell signalling	up-regulation	3.17
11	T_5	PDCD2L	regulation of cell signalling	up-regulation	2.43
8	T_4	SLC26A2	transport of molecules	down-regulation	-3.38
11	T_3	SCN7A	transport of molecules	down-regulation	-2.70
11	T_3	SLC2A3	transport of molecules	down-regulation	1.04
3	T_5	IPO5	transport of molecules	up-regulation	1.69
3	T_5	RANBP6	transport of molecules	up-regulation	1.69
